# Examining the Water–Polymer Interactions in Non-Isocyanate Polyurethane/Polyhedral Oligomeric Silsesquioxane Hybrid Hydrogels

**DOI:** 10.3390/polym16010057

**Published:** 2023-12-23

**Authors:** Izabela Łukaszewska, Artur Bukowczan, Konstantinos N. Raftopoulos, Krzysztof Pielichowski

**Affiliations:** Department of Chemistry and Technology of Polymers, Cracow University of Technology, ul. Warszawska 24, 31-155 Kraków, Poland; artur.bukowczan@pk.edu.pl (A.B.); konstantinos.raftopoulos@pk.edu.pl (K.N.R.)

**Keywords:** non-isocyanate polyurethanes, POSS, hydrogels, water absorption, glass transition

## Abstract

Non-isocyanate polyurethane (NIPU) networks physically modified with octa(3-hydroxy-3-methylbutyldimethylsiloxy)POSS (8OHPOSS, 0–10 wt%) were conditioned in environments of different relative humidities (up to 97%) to study water–polymer interactions. The equilibrium sorption isotherms are of Brunauer type III in a water activity range of 0–0.97 and are discussed in terms of the Guggenheim (GAB) sorption model. The study shows that the introduction of 8OHPOSS, even in a large amount (10 wt%), does not hinder the water affinity of the NIPU network despite the hydrophobic nature of POSS; this is attributable to the homogenous dispersion of POSS in the polymer matrix. The shift in the urethane-derived carbonyl bands toward lower wavenumbers with a simultaneous shift in the urethane N-H bending bands toward higher wavenumbers exposes the breakage of polymer–polymer hydrogen bonds upon water uptake due to the formation of stronger water–polymer hydrogen bonds. Upon water absorption, a notable decrease in the glass transition temperature ($T_{g}$) is observed for all studied materials. The progressive reduction in $T_{g}$ with water uptake is driven by plasticization and slaving mechanisms. POSS moieties are thought to impact slaving indirectly by slightly affecting water uptake at very high hydration levels.

## 1. Introduction

**Figure 1:**
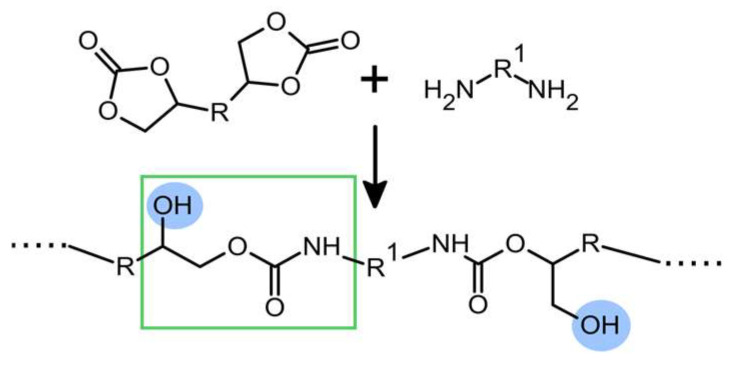
Scheme of polyhydroxyurethane formation by aminolysis of cyclic carbonate. Hydroxyl groups are highlighted in blue and hydroxyurethane moiety is shown in the green rectangle.

Non-isocyanate polyurethanes (NIPUs) have attracted a great deal of attention in recent years, and numerous studies have been conducted on the development of new synthesis routes [1,2,3]. The most promising and the most widely applied is the aminolysis of cyclic carbonate, which leads to the formation of polyhydroxyurethanes (PHUs). One of the most important differences between PUs and PHUs is the formation of additional hydroxyl groups in close vicinity to the urethane moiety (Figure 1). Such hydroxyurethane groups strongly affect the interaction between PHUs and organic solvents and cause a relatively high affinity of PHUs towards water [4]. Thus, PHUs, if not modified by the introduction of other, less polar moieties, such as amide groups [5], exhibit significantly higher water absorption than PUs [6,7,8,9,10,11]. This makes polyhydroxyurethanes suitable for applications as hydrogels [12,13] and absorbents [14].

**Figure 2:**
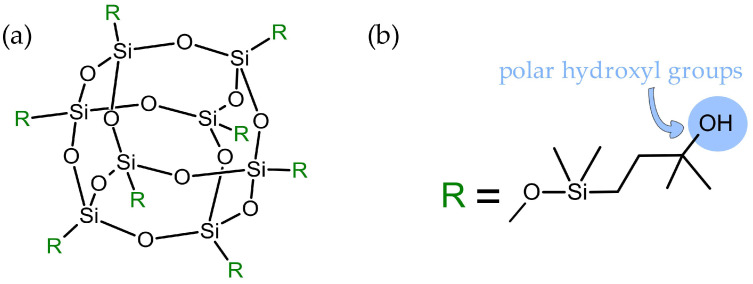
General structure of closed-cage substituents (R) might be of the same chemical nature or they can vary (**a**), and structure of the substituents in the octa(3-hydroxy-3-methylbutyldimethylsiloxy) POSS used in this study (**b**).

As the modification of polyurethanes leads to advantageous results, the impact of different types of additives on the properties of NIPUs has been studied [15,16,17,18,19], including polyhedral oligomeric silsesquioxanes (POSS) [20,21,22,23,24]. POSS consists of a group of hybrid (organic/inorganic) molecules that are characterized by a well-defined silica cage core that is usually functionalized by reactive or non-reactive substituents (Figure 2) [25].

The characteristics of the substituents affect the type of interaction between POSS and the polymer matrix [26,27,28]. POSS may be introduced chemically or physically into the polymer structure [29,30,31,32]. Chemical introduction is feasible if substituents attached to the POSS cage are equipped with reactive groups (in the given system) that would allow for covalent bonding with the chain [33,34]. The physical introduction of POSS occurs when POSS-bearing inert groups are introduced into the polymer matrix to form a (nano)composite [35]. Generally, the physical blending of POSS in a polymer matrix is associated with partial agglomeration of POSS since there is a high surface free energy and a high affinity of nanoparticles [35,36,37].

Subsequently, the type of POSS and the method of its introduction into the polymer matrix will influence the properties of the resultant hybrid composites. POSS may positively influence biocompatibility [38,39], flame retardancy [40,41], mechanical properties [37,38,42], etc. However, to achieve the desired effect, proper selection of POSS and the polymer matrix is crucial [28].

Recent studies on NIPU modification with POSS focus mainly on systems where POSS is introduced covalently into the polymer chain [3,20,21,22,23,24,43,44,45,46,47,48,49]. The impact of chemically incorporated POSS in such systems on mechanical and thermal properties has been widely studied. However, little attention has been paid to NIPU/POSS physical blends, and even less attention has been paid to determining the impact of POSS on the hydration properties of NIPUs, even though the high water absorption seems to be a dominant feature for this kind of material. Taking into account the biocompatibility of POSS [50,51] and the possible application of NIPUs in biomedical fields (e.g., in wound treatment) [52,53], shedding light on the influence of POSS on water–polymer interactions in such systems is interesting from the point of view of polymer and material science. It should be noted that although water–polymer interactions attract a lot of attention due to their obvious significance in biotechnology [54,55], more often than not, the studies are performed in solutions, i.e., in an environment of abundant water. Less attention has been paid to the initial stages of hydration and the interaction of the polymer with humid environments, as opposed to direct interaction with water in a solution. However, this low-hydration regime is interesting from the fundamental science point of view as well as from the application side, i.e., in the case of mucosal drug delivery.

In a previous article, we studied hydrophilic NIPU networks physically modified with octa(3-hydroxy-3-methylbutyldimethylsiloxy)POSS (8OHPOSS) with respect to their morphology, hydrogen bonding, and glass transition in a dry state [56]. We found in this previous work that POSS disperse excellently in the NIPU network, possibly thanks to the affinity of the hydroxyl groups on POSS vertices with the polymeric chain. Changes in the glass transition were attributed to competition between mechanisms accelerating or decelerating mobility. Moreover, we studied the interaction of the systems with water in abundance and observed that POSS enhances water absorption in a saline solution.

Since our previous study showed that POSS/NIPU networks may act as hydrogels, we now focus our attention on studying polymer–water interactions in such systems in detail. POSS is a promising additive in biomaterials [57] and, therefore, its influence on water–polymer interactions is paramount from the point of view of applications. In the work at hand, we extend the research on the very same materials [56] to their hydration properties in humid environments. Polymer–water interactions are studied by conditioning materials in a range of progressively increasing relative humidity environments. Materials are studied with respect to their water uptake and its influence on hydrogen bonding and molecular mobility. The GAB sorption model is used to determine the monolayer capacity and interaction parameters.

## 2. Materials and Methods

### 2.1. Materials

The materials studied in this work, i.e., a series of NIPUs networks physically modified with octa(3-hydroxy-3-methylbutyldimethylsiloxy)POSS (8OHPOSS), are the same as those described in Ref. [56]. Their synthesis, structure, and micromorphology are described in detail in this previous publication. Briefly, poly(ethylene oxide)-based cyclic carbonate (PEO-CC, Specific Polymers, Castries, France) of M_w_~700 g/mol was mixed with hyperbranched polyethyleneimine (PEI, Sigma-Aldrich, Darmstadt, Germany) of M_w_~650 g/mol to form a polyhydroxyurethane network. The reaction was carried out at 50 °C in dimethylacetamide (DMAc, Pol-Aura, Zabrze, Poland) using 1,5,7-Triazabicyclodec-5-ene (TBD, Sigma-Aldrich, Darmstadt, Germany) as a catalyst. 8OHPOSS (Hybrid Plastics, Hattiesburg, MI, USA) was added at the beginning of the reaction, along with all other components.

Phosphorus pentoxide (P_4_O_10_) and all salts listed in Table 1 were supplied by Chempur (Piekary Śląskie, Poland). Salts were added to distilled water to prepare saturated solutions to control the humidity.

### 2.2. Specimen Conditioning

**Figure 3:**
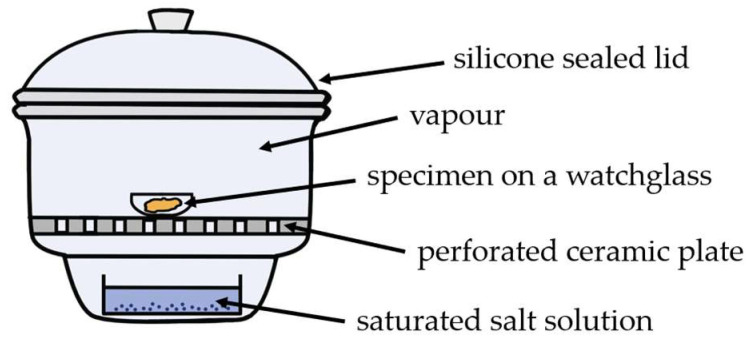
Schematic representation of specimen conditioning in a desiccator over saturated salt solutions.

Specimens of ~250 mg were first dried at 80 °C for 48 h under vacuum and subsequently conditioned in a desiccator over P_4_O_10_ in order to remove any residual water [58]. The mass values obtained after the specimens reached equilibrium in P_4_O_10_ were considered the masses of dry materials ($m_{dry}$). Subsequently, the samples were conditioned at progressively increasing levels of relative humidity (*rh*) (Figure 3) provided by saturated aqueous salt solutions (Table 1) [59]. The procedure was carried out at 25 °C. The equilibrium state was assumed when no further change in specimen mass was recorded between measurements with a two-day time interval. The time needed for reaching the equilibrium state was approximately 2–3 weeks per hydration level.

After mass equilibrium was reached at the given *rh* level, the specimens were weighed on an analytical scale with a precision of 0.1 mg. Water uptake (WU) was calculated as in Equation (1):

(1) $$WU=\frac{m_{hydrated}-m_{dry}}{m_{dry}}100\%$$

where $m_{dry}$ is the mass of dry specimens (after conditioning over phosphorus pentoxide) and $m_{hydrated}$ is a mass of hydrated specimen at a given rh level.

Alongside these samples, smaller pieces (~5–6 mg) were placed in perforated aluminum pans for DSC experiments (Section 3.3) and pieces of mass of ~300 mg, which were used for the FTIR experiments (Section 3.2).

### 2.3. Analysis of Equilibrium Sorption Isotherms

To describe equilibrium sorption isotherms, the Guggenheim–Anderson–de Boer (GAB) model was fitted to the data. The GAB model constitutes a generalization of the BET model describing the sorption of the materials based on the theory of the formation of monolayers and further layers, the so-called multilayers. The GAB model (Equation (2)) takes into consideration the difference in interactions between monolayers and multilayers, as well as that between multilayers and “bulk” water. Therefore, the GAB model introduces a third parameter K that is associated with the energy of the multilayer–bulk water interaction, while the C parameter in the GAB model is a constant correlated with the monolayer–multilayer interaction energy [60,61].

(2) $$M_{w}=\frac{M_{0}CKa_{w}}{\left(1-{Ka}_{w})(1+CKa_{w}-Ka_{w}\right)}$$

$M_{w}$—equilibrium water content in relation to dry mass

$M_{0}$—monolayer (first sorption layer) moisture content

C—energy constant logarithmically related to the difference in water molecule potential in the monolayer and multilayer

K—energy constant logarithmically related to the difference in water molecule potential in the multilayer and in the “bulk”

$a_{w}$—water activity

### 2.4. Fourier Transform Infrared Spectroscopy (FTIR)

Fourier transform infrared (FTIR) spectroscopy analysis was carried out using a Thermo Scientific Nicolet iS5 spectrometer equipped with a diamond prism iD7 ATR Accessory (Waltham, MA, USA). Spectra were recorded in the wavenumber range of 4000–400 cm^−1^ with a data gap of 0.428 cm^−1^ and a scanning resolution of 4 cm^−1^.

### 2.5. Differential Scanning Calorimetry (DSC)

Differential scanning calorimetry measurements were conducted with an argon-purged differential scanning calorimeter Mettler Toledo 823e (Columbus, OH, USA), cooled by liquid nitrogen. The DSC curves were recorded in the temperature range of −100 °C to 30 °C with a 10 K/min heating/cooling rate. The glass transition temperature ($T_{g}$) values were calculated at the midpoint of the endothermic step. Due to the absorption of water, the masses varied through hydration levels and stayed in the general range of 5–9 mg. Samples for DSC measurements (one sample per material) were prepared in an aluminum pan with a perforated lid. DSC samples were conditioned alongside those for water vapor absorption measurements. The DSC measurements were conducted after the equilibrium state was reached.

## 3. Results and Discussion

NIPU networks at hand upon immersion in an aqueous medium exhibit water absorption in the range typical for hydrogels, and we have observed this kind of behavior in our previous work [56]. To study polymer–water interactions in such a system in detail, the changes in physicochemical properties of the NIPUs were monitored as a function of the environmental relative humidity (water vapor absorption).

### 3.1. Water Uptake and Equilibrium Sorption Isotherms

**Figure 4:**
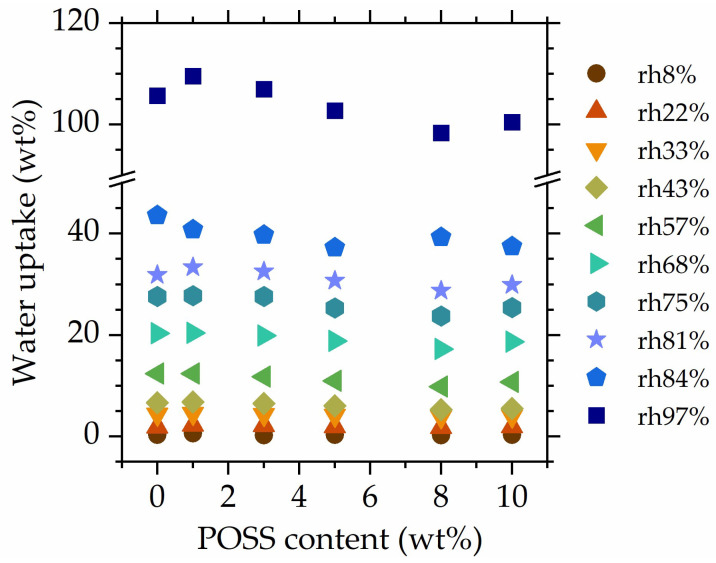
Water uptake as a function of the POSS content at all studied rh levels.

Figure 4 shows the water uptake as a function of POSS, recorded at all *rh* levels. With increasing relative humidity, water uptake differences between *rh* levels become more pronounced, showing that already-absorbed water increases the materials’ affinity towards water vapor. At low *rh* levels, no differences in the water absorption of specimens with different POSS contents are observed. At high relative humidities (>68%), slight differences emerge; however, their nature is not monotonous, and the observed differences in water uptake are rather small. Therefore, it can be concluded that 8OHPOSS does not hinder the water absorption of the studied materials, even at high POSS contents (10 wt%). This might originate from hydration sites still being accessible to water molecules, even at high POSS loadings. The addition of POSS of course reduces the number of NIPU-originating hydration points per unit mass; however, the effect is small (~10%) and likely compensated to some extent by the introduction of hydroxyl groups residing on the vertex groups of POSS. We will further follow this point with a formal analysis of equilibrium sorption isotherms.

**Figure 5:**
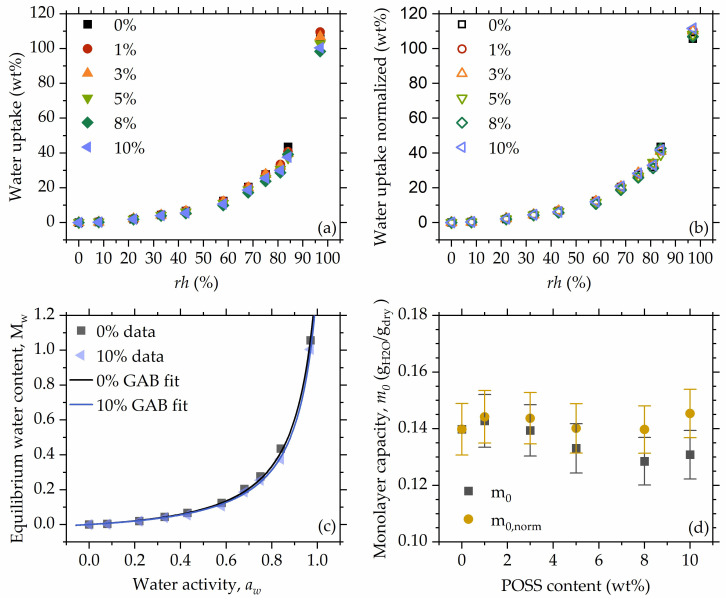
Equilibrium sorption isotherms (**a**), sorption isotherms with water uptake normalized to polymer mass (**b**), fitted curves shown for 0% and 10% as examples (**c**), monolayer capacity obtained from the model fitting as calculated ($m_{0}$), and normalized to polymer content ($m_{0},norm$) (**d**).

Equilibrium sorption isotherms (ESI) (Figure 5a) show a Brunauer type III form [62], which is observed mainly for systems such as biopolymers, microporous polymeric adsorbents, and water-soluble solids [63,64,65], and suggests sorption through the capillary condensation mechanism [66]. Interestingly, ESI of type III is often observed for conventional and non-isocyanate polyurethanes [5,67,68]. Slight differences in water uptake (Figure 5a) vanish when water absorption is normalized to the polymer mass (Figure 5b), which supports the previous statement regarding the lack of POSS influence on the water absorption, despite the fact that silica fillers could decrease water absorption (g water/g polymer) in composite materials [69].

To characterize materials in terms of monolayer capacity, the Guggenheim–Anderson–de Boer (GAB) model was fitted (Figure 5c). Preliminary fits did not show any significant differences in the C and K values, while significant covariances were observed. This stability suggests that the apparent interaction strength between the polymer chain and water situated in the monolayer is constant through the whole composition range, i.e., POSS does not influence polymer–monolayer water interactions. To increase the statistical significance of the monolayer capacity and interaction parameters, a second fit was performed simultaneously for all sorption datasets, assuming C and K values as constant throughout the POSS content range. The resulting values for C and K were 0.63 ± 0.071 and 0.918 ± 0.004, respectively. The C parameter exhibits a rather low value compared to the values reported in the literature for similar systems [67,68]; however, it remains in the range characteristic for Brunauer type III isotherms [70]. The K parameter is close to 1.0 and of the value is usually reported for the materials exhibiting Brunauer type III behavior [67,68]. The closer the K parameter is to unity, the more similar the behavior of the formed multilayers is to the behavior of liquid water [71].

Monolayer capacity ($m_{0}$) showed a slightly decreasing trend with increasing POSS content; however, after normalizing it to the polymer mass in the material, the values are stable throughout the entire composition range (Figure 5d). This points to the assumption that the absorbed water is located on the polymer chain, especially at lower water uptakes, and not around POSS molecules. Taking also into account that there is no significant change in the energetic parameters C and K, it may be concluded that 8OHPOSS does not hinder access of water to hydration sites and it does not “block” any of them. Although in [56] we observed some attachment of POSS on the polymer chain by hydrogen bonds, this does not hinder the attachment of the more mobile and more polar water molecules to access the hydration sites. The obtained values of monolayer capacity are around 14 g per 100 g of dry material, which is more than 25 times higher than in cellulose-based hydrogels studied by Filip et al. [72], 3.5 times higher than for acrylate hydrogels reported by Ferrer et al. [73], and 1.5 times the value reported for polyacrylamide-based superporous hydrogel composites reported by Mittal et al. [74].

### 3.2. Water Influence on Carbonyl Region—FTIR

**Figure 6:**
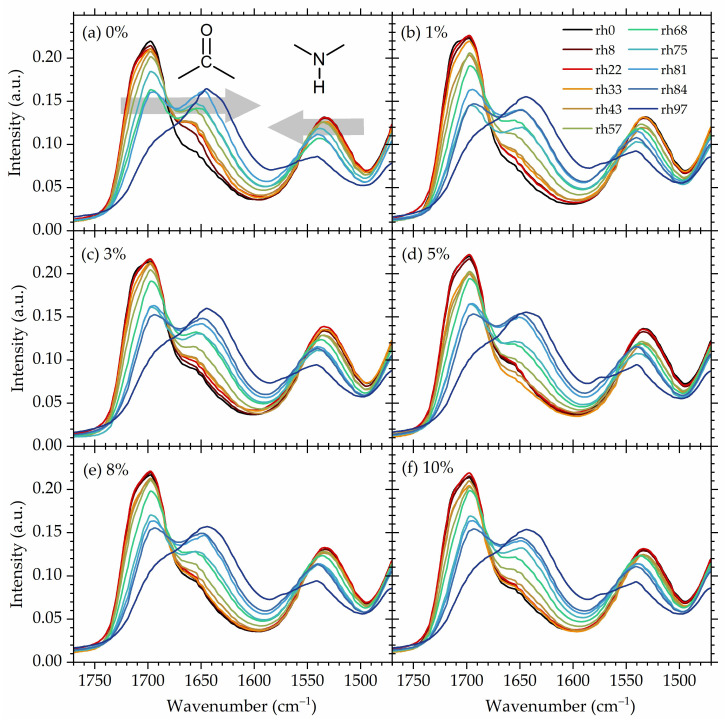
FTIR spectra in the urethane region of all studied NIPUs at all hydration conditions. Spectra of rh 0% are taken from Ref. [56]. (**a**–**f**) spectra for different specimen as indicated in the plot. Arrows indicate the shift of the band with increasing hydration.

Hydroxyurethane moieties are the most polar sites in the chains of studied NIPUs, and therefore they consist of the strongest (primary) hydration sites. Thus, one should expect that absorbed water molecules are most likely to attach to those moieties, prioritizing carbonyl as the most polar region of the hydroxyurethane group. The carbonyl stretching region is known to be sensitive to hydrogen bonding and usually consists of the region most commonly studied in terms of quantitative and/or qualitative analysis of HBs in polypeptides [75,76,77], polyureas [78,79], polyamides [80,81,82], conventional polyurethanes [83,84,85], and non-isocyanate polyurethanes [58,86,87]. Figure 6 shows, for all materials under investigation, FTIR spectra in the region corresponding to the stretching vibration of urethane-derived carbonyl (1630–1730 cm^−1^) and the bending vibration of urethane-derived NH groups (1500–1580 cm^−1^) at all studied hydrations. The spectra were recorded after equilibrium at a given hydration level was reached.

The carbonyl stretching region exhibits a complex nature with three main component bands overlapping, as is also usually observed for conventional polyurethanes [88,89,90]. Here, the components in the carbonyl stretching region are as follows (locations given in relation to the spectra of dry materials): 1720 cm^−1^ corresponding to the free carbonyls (i.e., non H-bonded), 1700 cm^−1^ corresponding to the weakly H-bonded ones, and 1660 cm^−1^ corresponding to the strongly H-bonded carbonyls (e.g., forming two or more hydrogen bonds) [58].

Upon water uptake (upon an increase in the rh level), the free C=O component gradually vanishes, while two other components shift towards a lower wavenumber (redshift). The shift towards a lower wavenumber of stretching modes indicates the formation of new hydrogen bonds and/or the strengthening of the already existing HBs [91,92,93]. In this study, the red shift in C=O stretching modes upon water absorption is related to the formation of water–polymer hydrogen bonds that either replace the polymer–polymer HBs (breakage of C=O⋯H-N hydrogen bonds and formation of C=O⋯H_2_O resulting from competition between water O-H and amide N-H for C=O acceptor sides [94]) or are formed as additional hydrogen bonds. In both cases, the resulting hydrogen bonding of C=O becomes stronger since hydroxy moieties form with carbonyl stronger hydrogen bonds in comparison to NH groups. The stronger hydrogen bonding of C=O⋯H-O compared to C=O⋯H-N results from the higher polarity of the OH bond compared to the NH bond [95]. The strength of hydrogen bonds influences the strength—the bigger the shift, the stronger the hydrogen bonding [96,97,98,99].

Simultaneously, the band associated with urethane NH bending gradually shifts towards higher wavenumbers (blue shift) [100], suggesting that polymer NH groups are also engaged in new, stronger interactions with water [5,101]. It is commonly observed that upon the formation of hydrogen bonds and/or the strengthening of existing ones, bands corresponding to the bending vibrations shift in the direction opposite to that of the stretching bands, i.e., towards higher wavenumbers (blue shift) [102,103,104,105,106,107].

**Figure 7:**
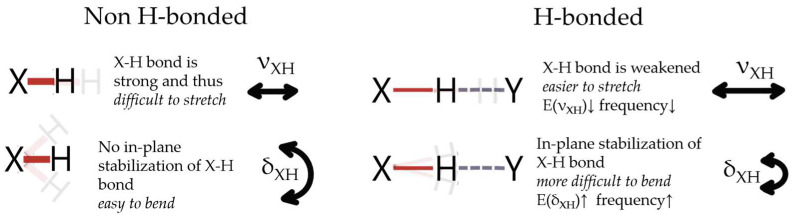
Schematic representation of the opposite shift-wise response of stretching and bending modes to the formation/strengthening of hydrogen bonds. Details in text.

The above originates from the inversely proportional relation between the stretching mode frequency and the bending mode frequency, that is, the lower the stretching mode frequency, the higher the bending mode frequency [105]. Upon the formation of X-H⋯Y hydrogen bonds, the X-H bonds undergo elongation (usually explained by electrostatic interaction or hyperconjugation), which causes stronger polarization of the X-H bond and leads to increased interaction with H⋯Y [95]. This elongation results in the weakening of the X-H bond due to the strengthening of H⋯Y [108]. The weakening of X-H enables stretching of this bond at a lower energy cost, and thus the shift in stretching modes towards lower wavenumbers is observed. Simultaneously, in the formed X-H⋯Y, stabilized complex forces of the X-H bond and H⋯Y interaction seem to promote the positioning of the X-H bond in the plane and therefore out-of-plane bending modes are more energetically expensive. Since the wavenumber is directly proportional to the energy [109], a shift toward a higher wavenumber is observed for the bending modes upon hydrogen bond formation. The opposing behavior of the stretching and bending modes upon HB formation is shown schematically in Figure 7. The above is consistent with the reported blue shift of the water bending modes upon the formation of a more dense hydrogen-bond network [110].

**Figure 8:**
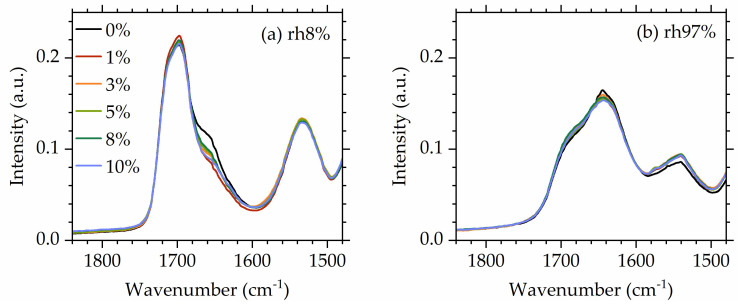
Comparative FTIR spectra in the urethane region for all studied NIPUs at (**a**) a low humidity (8%) and (**b**) a high humidity (97%).

Turning our attention to the influence of POSS on hydrogen bonding (Figure 8), it is interesting to observe that at low hydrations (rh 8%), POSS has some influence on the strength of the component of the carbonyl band assigned to strongly bonded carbonyls. This is on par with what was observed for dry materials [56]. However, at high hydrations, spectra of all materials coincide, indicating that POSS now does not play any significant role in hydrogen bonding of the carbonyl bands, presumably because water now dominates the behavior of the system.

As shown in Section 3.3, the replacement of polymer–polymer C=O⋯H-N hydrogen bonds with polymer–water C=O⋯H-O bonds increases the system’s mobility despite the increased strength of hydrogen bonding since now polymer forms HBs with small and mobile water molecules; as a result, a decrease in $T_{g}$ values upon water uptake is observed.

### 3.3. Influence of Absorbed Water on Glass Transition Temperature

**Figure 9:**
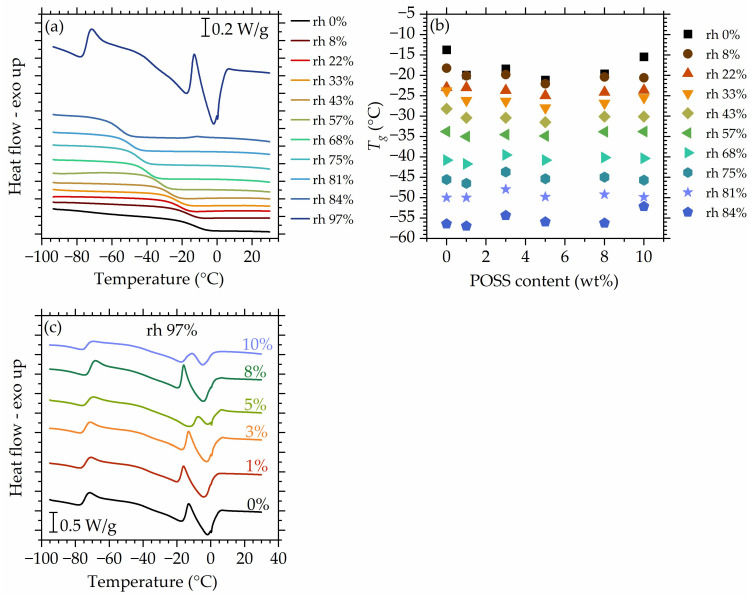
DSC curves recorded for the matrix at all studied hydrations. (**a**) Glass transition temperature as a function of the POSS content for materials studied at rh values up to 84% (**b**), DSC curves recorded for all studied materials after conditioning at rh 97% (**c**). Data for dry materials are taken from Ref. [56].

The DSC profiles recorded for the matrix after conditioning at progressively increasing hydration levels (Figure 9a) up to *rh* 84% contain only a glass transition step and show a gradual decrease in $T_{g}$ values. This is a very common phenomenon and results from increasing water uptake—water acts as a plasticizing agent that increases molecular mobility of the system and, therefore, reduces $T_{g}$. However, the mechanisms that drive it are more complicated. In the following, we will discuss them in detail for the system at hand. It is interesting to note that XRD experiments showed no signs of crystallinity in the composites upon hydration. Hence, we believe that the system is still homogenous at high hydrations. It is possible for physically introduced fillers to agglomerate when the mobility of the polymer increases. Here, however, we have no indication of such behavior, which might be attributed to molecules of absorbed water acting as a barrier that prevents POSS agglomeration.

Figure 9b shows $T_{g}$ values as a function of POSS content for all studied hydrations. With increasing hydration, a slight deviation of $T_{g}$ observed in the studied materials at a dry/low hydration state vanishes in the range of medium hydrations, as is expected, since POSS does not hinder water uptake or water–polymer interactions as shown earlier in this work. At *rh* 84%, a slight deviation in $T_{g}$ values is observed. This will be explained further in the text.

Figure 9c shows the DSC curves recorded for the materials after conditioning at *rh* 97%. At this *rh* level, the effects of ‘free’ water appear, which were not visible on the curves recorded for lower hydrations (Figure 9a). At −15 °C to −10 °C, cold crystallization [111] occurs and is followed by a melting just below 0 °C. The temperature of cold crystallization varies slightly and monotonously between materials. Interestingly, another endothermic event is present at around −70 °C, in the temperature range where the glass transition of the system would be expected. The origin of this event is unclear at this point. A plausible hypothesis is that it reflects a low-temperature cold crystallization of water, triggered by the glass transition of the system, now masked by the exothermic event.

**Figure 10:**
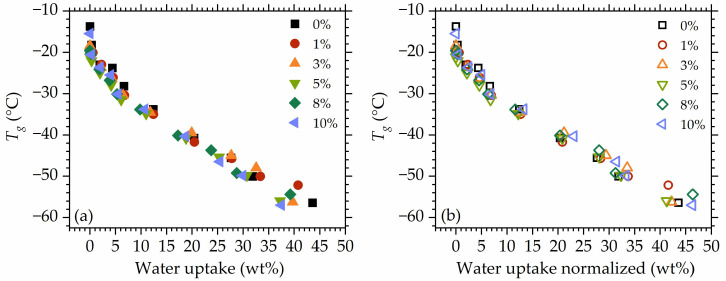
$T_{g}$ as a function of water uptake (**a**) and water uptake normalized to the polymer mass (**b**).

Plotting $T_{g}$ versus water uptake (Figure 10a) and normalized water uptake to the polymer mass (Figure 10b) show that the differences between mobility in the studied materials occur rather at the dry/low hydrated state. The highest deviations are visible at *rh* 87%. Moreover, at the initial hydration stages (at low water uptakes), a very steep decrease in $T_{g}$ is observed, despite the low water content in the materials.

**Figure 11:**
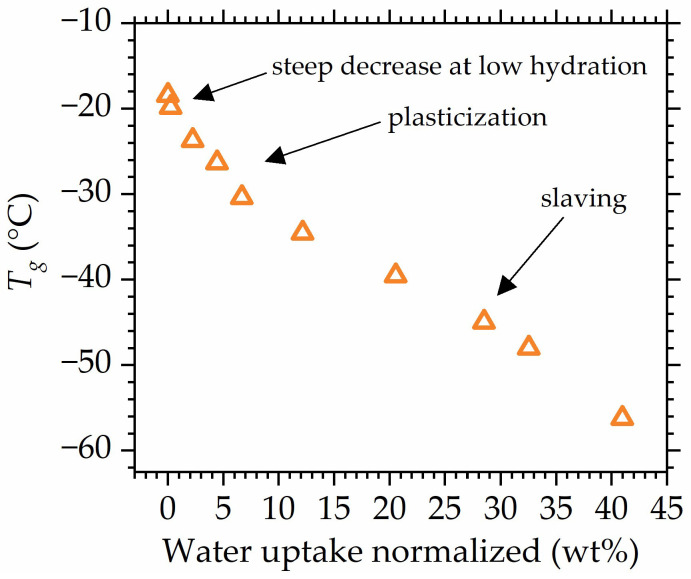
$T_{g}$ as a function of water uptake with changes in slope assigned to mechanisms (plasticization, slaving) driving $T_{g}$ decrease. Data for composite with 3 wt% POSS are shown as an example, all other studied materials exhibit the same behavior.

Looking closely at the change in $T_{g}$ values with water uptake (Figure 11), it can be concluded that at least two mechanisms drive the reduction in $T_{g}$, as two main different changes in slope might be distinguished. $T_{g}$ reduction is often described as plasticization; however, the latest studies use the term plasticization to describe only one of the mechanisms that drive the decrease in $T_{g}$ in hydrated polymers [56,57]. Therefore, to avoid terminology conflict, the decrease in the glass transition will be referred to as $T_{g}$ reduction, while *plasticization* will be used to describe the water-driven increase in polymer mobility that occurs due to the increase in the flexibility of the polymer chain [112].

Plasticization is observed in low to medium hydrations; however, at high relative humidities, another effect seems to impact the mobility of polymer–water systems. At high hydrations, when the amount of absorbed water is high enough for bulk water to be formed, the water gains its own mobility. As a result, water mobility now determines polymer mobility in a sense that the dynamics of bulk water and dynamics of polymer are now coupled and the observed glass transition of the systems is a result of cooperative motion between two components that possess their own dynamics [113]. Therefore, water possessing it own mobility drives $T_{g}$ reduction in a different manner than in the case of plasticization, which is seen as a pronounced change in slope in the data points (Figure 11). The above is referred to as *slaving*, since polymer mobility is “slaved” by bulk water [112,114,115,116,117].

The fact that in the range of the medium hydration levels, the dependency is the same for all studied materials shows that POSS does not influence the plasticization of the polymer matrix. However, at high hydrations, where slaving is the dominant phenomenon, the deviation between specimens’ behavior becomes more pronounced, suggesting that POSS slightly impacts the slaving mechanism. This impact might result from the hindering of water mobility by the dispersed POSS molecules that possess hydrophobic silica cages. Therefore, as shown in Figure 6, weak differences between $T_{g}$ values of the specimens appear at high hydrations. Those deviations exhibit a different nature from the ones spotted for dry/low-hydrated materials, which suggests that they are of a different origin. Thus, the most plausible explanation is the disclosure of POSS–water interactions at high hydrations (high water uptakes) that hinder water mobility. The above might be related to the fact that at very high water uptake, most of the free volume in the material is already hydrated, and now water molecules attach to the hydroxy moieties at the ends of POSS substituents.

## 4. Conclusions

POSS-modified NIPU networks exhibit a high affinity for absorbing moisture from the air that allows for water uptake typical of hydrogels even when the materials are not immersed in water (water uptake up to 115 wt% in *rh* 97%). POSS, despite its heavy hydrophobic silica cage, does not hinder water absorption capacity, even at high loadings (10 wt%)—it shows that 8OHPOSS, due to its endcapping OH polar groups, is able to disperse homogenously in the matrix and that this homogeneity is retained upon water absorption. The above shows that 8OHPOSS might be successfully used as filler for the modification of other properties, without a negative influence on materials’ affinity towards moisture/water absorption—normalizing water uptake to the polymer mass yields the same values for all studied composites. Moreover, according to the fitted GAB model, water absorption capacity is not inhibited by POSS.

Absorbed water causes cleavage of polymer–polymer and polymer–POSS hydrogen bonds due to the formation of stronger water–polymer bonds, as indicated by a red shift in the urethane-derived C=O band and the simultaneous blue-shift in urethane NH bending modes. The above results in a reduction in $T_{g}$ values upon hydration. The $T_{g}$ reduction occurs according to two mechanisms—plasticization and slaving. Plasticization occurs at low to medium hydrations, where absorbed water promotes the elasticity of the polymer chain. Slaving is observed at higher hydrations, where the amount of absorbed water is high enough to cause the formation of bulk water that possesses its own dynamics.

The 8OHPOSS does not influence the changes in $T_{g}$ as a function of water uptake or relative humidity, which is to be expected since the introduced POSS does not hinder the water absorption capacity and polymer–water affinity in the entire composition range.

The DSC curves recorded for the specimens conditioned at the highest studied relative humidity level (*rh* 97%) show effects related to the so-called “free” water, mainly cold crystallization around −15 °C followed by melting slightly below 0 °C, and the third effect at −70 °C of yet unclarified origin. To explain the origin of this third phenomenon, further studies focused on the mobility of water in confinement are required.

## Figures and Tables

**Table 1:** Salts used to prepare solutions for conditioning materials and corresponding relative humidity levels of their saturated solutions [2].

Salt	Relative Humidity at 25 °C (%)	*rh* Designation in the Manuscript
Potassium hydroxide (KOH)	8.23 ± 0.72	8%
Potassium acetate (CH_3_COOK)	22.51 ± 0.32	22%
Magnesium chloride (MgCl_2_)	32.78 ± 0.16	33%
Potassium carbonate (K_2_CO_3_)	43.16 ± 0.39	43%
Sodium bromide (NaBr)	57.57 ± 0.40	57%
Potassium iodide (KI)	68.86 ± 0.24	68%
Sodium chloride (NaCl)	75.29 ± 0.12	75%
Potassium bromide (KBr)	80.89 ± 0.21	81%
Potassium chloride (KCl)	84.34 ± 0.26	84%
Potassium sulfate (K_2_SO_4_)	97.30 ± 0.45	97%

## Data Availability

Data will be available on request.
